# Sensory Attributes of Buckwheat Jelly (Memilmuk) with Mung Bean Starch Added to Improve Texture and Taste

**DOI:** 10.3390/foods10112860

**Published:** 2021-11-18

**Authors:** Yongseok Kwon, Jihye Ryu, Seyoung Ju

**Affiliations:** 1 National Institute of Agricultural Sciences, 166 Nongsaengmyeong-ro, Wanju-gun 55365, Korea; selenium2012@korea.kr (Y.K.); jhryu13@korea.kr (J.R.); 2 Major in Food Science, College of Biomedical and Health Science, Konkuk University, Chungju 27478, Korea

**Keywords:** descriptive sensory analysis, consumer acceptability, buckwheat jelly

## Abstract

Buckwheat contains more essential proteins, dietary fiber, vitamins, minerals, and diverse phytochemicals than wheat and rice. The aims of this study are to develop the descriptive sensory attributes and evaluate the consumer acceptability of six buckwheat jellies (memilmuk) with added mung bean starch and to analyze the relationship between their descriptive sensory attributes and consumer acceptability. Statistical analyses were performed by one-way analysis of variance (ANOVA), principal component analysis (PCA), and partial least squares regression (PLSR). A total of 18 sensory attributes of buckwheat jelly, including appearance (brown, brightness, and roughness), odor/aroma (soymilk smell, grain smell, red bean porridge smell, and buckwheat tea smell), flavor or taste (savory flavor, plain taste, buckwheat taste, sweet taste, salty taste, and umami), and texture (squashed, dry, smooth, elasticity and stickiness) were developed. Consumer acceptability tests of six buckwheat jellies were conducted by 93 consumers evaluating for color, smell, savory taste, aftertaste, harmony with the sauce, overall liking, and would recommend or try again. Buckwheat jelly with 25% of mung bean starch (BJ_916) was the most favorable jelly sample among the six samples. All attributes except color, smell, and the savory taste of samples showed a significant difference (*p* < 0.001). BJ_916 showed a close relationship with a grain smell, elasticity, red bean porridge smell, and sweet taste of descriptive attributes and also all attributes of consumer acceptability. The determination of sensory attributes and consumer acceptability of buckwheat jelly will help to improve sensory characteristics to fulfill consumer needs and desires. Furthermore, this current study will help facilitate the expansion of the buckwheat consumption market.

## 1. Introduction

Buckwheat (*Fagopyrum esculentum*) is a short-season crop belonging to the Polygonaceae family [1]. Buckwheat is called a pseudo-cereal because the utilization and nutritional value of buckwheat seeds are similar to those of wheat or oat as cereal grains. Buckwheat originally came from Central Asia and is now mainly cultivated in Asia and eastern Europe [2]. Buckwheat groats are commonly used for bread, cookies, and pancakes in western Asia and eastern Europe while buckwheat noodles and pasta have been eaten in eastern Asia and Italy [3]. In Korea, buckwheat flour is consumed to make noodles, pancakes, and jelly (muk) [4]. Buckwheat includes a higher protein content (14%) than that of rice (8.2%) [5]. Furthermore, it is a good source of protein because it contains more essential proteins (cysteine and methionine) compared to rice or maize [2].

The total dietary fiber of buckwheat is also significantly higher than common grains, such as rice and maize [5]. Moreover, it is rich in dietary fiber, vitamins, minerals, and diverse phytochemicals, such as flavonoids, tocols, and rutin [6,7]. These high functional components contribute to various health benefits; it has been found to decrease blood pressure, act as an antidiabetic, have antioxidant, antimicrobial, anticancer effects, and reduce risk of cardiovascular diseases [3,8,9,10,11,12,13]. Buckwheat is also helpful for people with celiac disease because buckwheat protein is gluten free [14].

Muk is a starch jelly that is a traditional Korean food made from grains, such as buckwheat, acorn, and mung bean, and has been consistently popular due to its nutritional profiles and peculiar texture (soft and chewy). It is produced by extracting starch from grains, boiling with water until thickened, and hardening to jelly [15]. Mung bean (*Vigna radiata*) is a legume mainly cultivated in Asia. Mung bean contains plenty of nutrients, such as proteins, vitexin, and isoflavone. It is currently used in plant-based meat and egg alternatives as well as soups, porridge, snacks, bread, and noodles [16]. In Korea, it is a popular food ingredient used in the form of sprouts and seeds, which are made into pancakes, jelly (muk), or seasoned sprouts (namul). Specifically, mung bean starch is a very good ingredient for jelly because of its excellent gel formation ability and high elasticity [17].

Recent studies on starch gel with buckwheat or mung bean were mainly focused on the efficacy of buckwheat or mung bean, such as its quality characteristics by adding functional ingredients. The studies included the effects of chitosan on shelf life and quality on buckwheat starch jelly [18], the effects of ginkgo nut powder on the quality characteristics and antioxidant activity of mung bean starch gel [19], and the effects on the quality characteristics of mung bean starch jelly with white lotus (*Nelumbo nucifera*) root powder [20]. Moreover, several studies have reported the rheological and textural properties of buckwheat bread and noodles for improving quality characteristics [4,21,22]. Previous studies on buckwheat jelly have mostly focused on the quality characteristics and bioactivities rather than organoleptic properties [23,24].

This study aims to evaluate the sensory properties and consumer acceptability of the jelly to improve the taste and texture of Korean traditional buckwheat jelly (memilmuk). We hope this study will help to develop healthy and consumer-favorable products with buckwheat and to contribute to the expansion of the market for buckwheat products.

## 2. Materials and Methods

### 2.1. Preparation of Buckwheat Jelly Samples

Table 1 presents the ingredients, sample code, and cooking methods used for the samples. The buckwheat flour (Hallasan area Cheotmaeul, Hallasan Co., Seogwipo, JeJu, Korea) harvested in Jeju-do, was purchased from a local online market because Jeju-do is the region with the highest buckwheat yield in Korea [25]. Other ingredients were salt (CJ Cheiljedang Co., Seoul, Korea), soybean oil (Sajo Co., Seoul, Korea), and mung bean powder (Gomine, Foodsynergy Co., Seoul, Korea).

This present study evaluated and compared sensory properties between pure buckwheat flour jelly and buckwheat flour jelly with 25% mung bean flour because commercial buckwheat flour for jelly contains 25% mung bean powder [26,27]. Samples in this study were processed with buckwheat flour and mung bean flour. The samples were six buckwheat jellies (memilmuk) with mung bean powder. The samples were named as follows: BJ_265 (no mung bean powder), BJ_153 (5% mung bean powder), BJ_870 (10% of mung bean powder), BJ_453 (15% of mung bean powder), BJ_335 (20% of mung bean powder), and BJ_916 (25% of mung bean powder). The manufacturing method for the jelly was based on “Buckwheat jelly (memilmuk) recipes” from Korean Traditional local food published by Rural Development Administration in Korea [28] and developed through experimental cooking and taste evaluations. The recipe is as follows: (1) Mix all the ingredients, filter twice through a sieve, and rest at room temperature for 15 min. (2) Put the mixture in a pot, put it on induction, stir at 180 °C for 5 min until it thickens, then lower to 120 °C and stir for 7 min. (3) Add cooking oil and stir for 5 min. Turn off the heat and lay flat on a square frame to harden. (4) After 5 h at room temperature, cut into 3 cm × 1 cm × 1 cm pieces.

Samples were put in a plastic bowl (7 cm in diameter × 3 cm in height) with 20 g (3–4 pieces) of jelly each and served to participants. The samples were coded with three-digit random numbers and presented using a Latin square design to minimize the carry-over effects [29], and spring water was served to allow participants to rinse their mouths between each sample.

### 2.2. Panel Selection and Training

Eight panelists (six females and two males, aged 20–40) with previous experience and interest in evaluating jelly products were selected. The basic screening tests, such as a basic taste test, flavor and aroma recognition test, and an intensity ranking test, were conducted 10 times to understand the basics of sensory evaluations by panelists [30]. Samples were presented in random order and labeled with three-digit random numbers according to the William’s Latin square design method [31,32,33]. The panelists were asked to swallow the samples after evaluating for a uniform tasting result.

During the first panel training for 120 min, the panelists tasted four samples, which were selected before the bench test, and generated sensory descriptive attributes for appearance, aroma/odor, taste/flavor, and texture/mouthfeel. This was performed through open discussions with panel leaders to establish terms and definitions of the attribute.

During the second training session (120 min), the panelists developed a refined set of sensory descriptive attributes using different products. Once the lexicon of interest was selected, various products were presented to the panel for the selection of reference standards.

For the third training session (120 min), chosen sensory descriptive attributes from the first and second training sessions were evaluated and confirmed after presenting selected and rated standard intensities of each attribute. The panelists gave ratings based on a 15-point intensity rating scale (0 = none; 15 = extremely strong) for each attribute.

During the fourth session (120 min), panelists practiced using selected standard samples for the final testing procedure. Pre-evaluation tests were conducted in individual booths equipped with white light systems using written evaluation sheets. The panelists then evaluated the real samples and compared the consistency of the scores. During the fifth training session (60 min), a preliminary test was investigated using standard samples to minimize rating variations between panelists. In each sensory test, panelists were served drinking water to eliminate any aftertaste and buckwheat jelly residues between samples. The panelists randomly evaluated three replicates of six samples in each sensory test. The sensory study protocol was reviewed and approved by Konkuk University’s Institutional Review Board (IRB approval number: 7001355-202011-E-126).

### 2.3. Development of a Lexicon for the Six Buckwheat Jellies

The panelists developed a lexicon with 18 descriptors to describe characteristics of appearance, aroma/odor, taste/flavor, and texture/mouthfeel. Regarding appearance, three descriptors (brown, brightness, and roughness) were developed. As aroma/odor and taste/flavor descriptors, soymilk smell, grain smell, red bean porridge smell, buckwheat tea smell, savory flavor, plain taste, buckwheat taste, sweet taste, salty taste, and umami were created. Five descriptors (squashed texture, dry texture, smooth texture, elasticity, and stickiness) as texture/mouthfeel descriptors were selected by the panelists. Furthermore, a preliminary test of sensory intensity was conducted to rate the relative intensities of the descriptors. A few panelists received supplementary training tests to minimize the deviation in intensity ratings. Table 2 shows the sensory descriptors, definitions, and physical standards of the six samples in this study.

### 2.4. Consumer Acceptance Test

The consumer panelists (*n* = 93, males: 20, females: 73, age: 20–60) were recruited to evaluate the consumer acceptability of the six buckwheat jelly samples. Each sample was presented to consumers using the same method as the descriptive analysis. Six randomized samples per session were presented to the consumer panelists using a randomized complete block design [31,34]. To minimize residual effects, spring water was provided between samples. Consumers were asked to rate their liking (color liking, smell liking, savory taste liking, aftertaste liking harmony with the sauce, overall liking, and likeliness to recommend and try again) using a nine-point hedonic scale (1 = dislike extremely to 9 = like extremely).

### 2.5. Statistical Analysis

The mean of the data was calculated using descriptive analysis. One-way analysis of variance (ANOVA) was performed to determine significant differences in sensory attributes and consumer acceptance between the six samples. A statistically significant difference was defined as *p* < 0.05. Duncan’s multiple range comparisons were performed by a post-hoc test if there was a significant difference at α = 0.05. Principal component analysis (PCA) was performed to obtain six samples and sensory representations of sensory attributes. Partial least squares regression (PLSR) analysis was also performed to identify associations between samples, technical attributes, and consumer acceptance. All statistical analyses were performed using SPSS (Statistical Package for Social Science) ver. 25.0 (IBM, Chicago, IL, USA) and SIMCA ver. 16.0 (Umetrics, Umea, Sweden).

## 3. Results and Discussion

### 3.1. Descriptive Sensory Analysis

Table 3 shows the intensities of the sensory attributes of six buckwheat jelly samples. Of the total of 18 sensory attributes, 8 attributes (Brown_A, Soymilk_O, Red Bean Porridge_O, Buckwheat Tea_O, dry texture, smooth texture, elasticity, and stickiness) showed a significant difference by sample (*p* < 0.05). The sensory attributes of Brown_A, Soymilk_O, Buckwheat tea_O, smooth texture, and stickiness were higher for jelly samples with a high buckwheat content. The samples with increased mung bean starch contents showed higher scores for red bean Porridge_O and elasticity than jelly samples with a high buckwheat content. Buckwheat proteins usually contain fewer prolamins and glutelins and more albumins and globulins than those of wheat and rye. Due to the composition of proteins, buckwheat flour has lower viscosity and elasticity compared to wheat [3,35]. However, mung bean flour has a high gel formation power, and mung bean jelly shows a smooth surface and high springiness because its texture is affected by the amount of amylose content and varieties of starch [36,37]. In the case of the dry texture attribute, the intensity score decreased in the buckwheat jelly samples with 5–20% of added mung bean starch, but it was a high score at 6.42 in the BJ_916 sample (sample with the highest mung bean starch content) with 25% mung bean starch added. Kim et al. [15] showed a similar result to this present study. They evaluated the quality characteristics of mung bean jelly (Cheongpomuk) with mung bean powder added (0, 25, 50, 75, and 100%). The results showed that the mung bean jelly with 25% mung bean powder had higher values of textural and sensory properties. The detailed confirmation of this result is necessary through assessing physicochemical properties in future studies.

### 3.2. Principal Component Analysis of the Six Buckwheat Jellies

**Figure 1:**
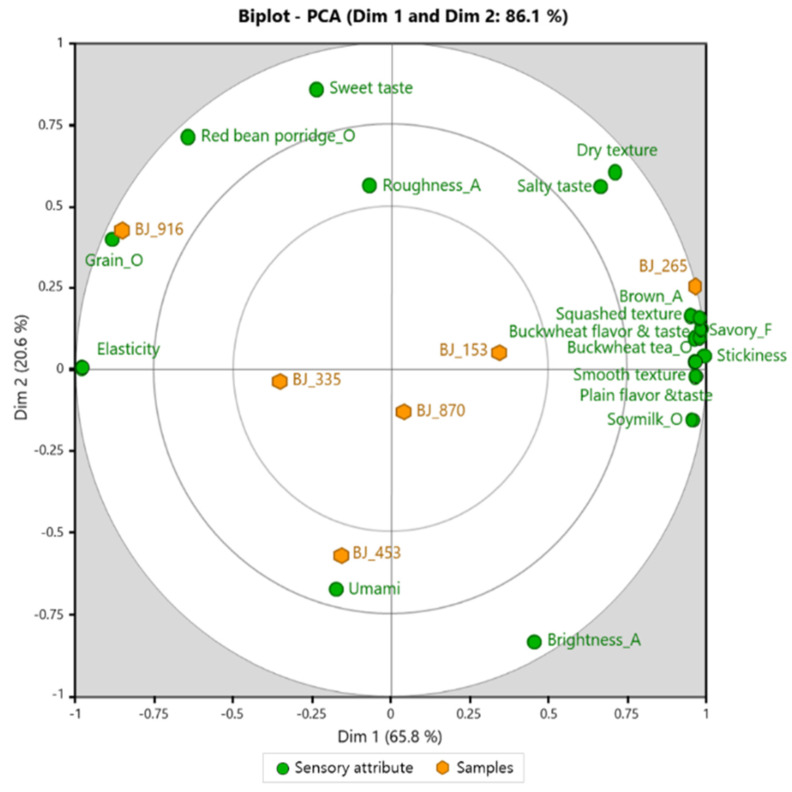
PC loadings regarding scores of the sensory attributes and the six buckwheat jelly samples evaluated by panels.

PCA was performed to provide a clear visualization of all sensory attributes in Figure 1. The PCA bi-plot accounted for 86.1% of the total variance with Dim 1 and Dim 2 explaining 65.8% and 20.6%, respectively. The sensory attributes contributing most to the right side of Dim 1 (i.e., *x*-axis) were Brown_A, Soymilk_O, Buckwheat tea_O, Savory_F, plain flavor and taste, buckwheat flavor and taste, squashed texture, smooth texture, and stickiness, and the BJ_265 (0% mung bean starch) sample was rated to be here. The attributes contributing to the left side of Dim 1 were elasticity and Grain_O, and the BJ_916 sample (25% mung bean starch) was rated to be here.

The attributes contributing to the positive side of Dim 2 (i.e., *y*-axis) were Red Bean Porridge_O and sweet taste, and the BJ_916 sample (25% mung bean starch) was rated to be here. The attributes contributing to the negative side of Dim 2 were umami, and the BJ_453 (15% mung bean starch) sample was rated to be here.

Overall, the BJ_916 (25% mung bean starch) sample was located on the left side of Dim 1, while samples with low mung bean starch contents were located in the center or to the right of Dim 1 (except for BJ_453). This result shows that elasticity tends to increase according to a lowering in the content of buckwheat flour and an increase in the content of mung bean powder.

### 3.3. Consumer Acceptance

The results of the consumer acceptability test for buckwheat jelly samples are presented in Table 4. A total of 93 consumers scored each sample for color, smell, savory taste, aftertaste, harmony with the sauce, overall liking, likeliness to try again, and intention to recommend. All attributes except color, smell, and savory taste of samples showed a significant difference (*p* < 0.001), and the acceptance attributes of the sample with the highest mung bean starch content (BJ_916: 25% mung bean powder) showed the highest scores among the six samples. Kim et al. [15] also showed that the overall acceptability of jelly with 25% mung bean powder was the highest among jelly with five different levels of mung bean powder (0, 25, 50, 75, and 100%). When we eat jelly, we expect to feel a smooth surface and a soft and elastic texture mouthfeel. These textural properties are closely related to the gel formation of starch. Greater gel formation power yields higher elasticity or springiness in terms of textural property. The gel formation of starch depends on the amylose and amylopectin ratio, starch chain length, type of starch, and degree of branching [36,37,38].

### 3.4. Relationships between Sensory Attributes and Consumer Acceptability

**Figure 2:**
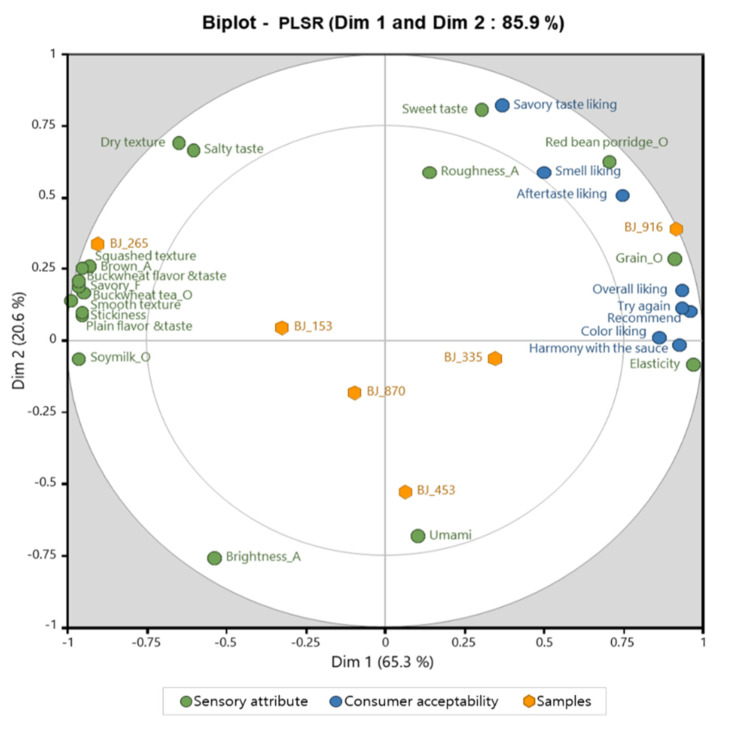
PLSR result indicating the association among sensory attributes, consumer acceptability and the six buckwheat jelly samples.

Figure 2 shows the relationships between sensory characteristics and consumer acceptability of the six buckwheat jelly samples. When PLSR analysis was conducted to determine the correlation between sensory characteristics and consumer acceptability, 85.9% of the total variance was explained by Dim 1 and Dim 2. PLSR analysis is a very suitable technique to analyze and visually summarize the correlation between two types of data [33,39,40]. BJ_916 (25% mung bean starch), which showed the highest score on consumer acceptability, was found to be near Dim 1 and had a close relationship with all attributes of consumer acceptability. For the results of descriptive sensory analysis, the BJ_916 sample (25% mung bean starch) was closely related to the attributes of Grain_O, elasticity, red bean Porridge_O, and sweet taste. Although the characteristics may have been different, a previous study analyzing the preference for rice-based snacks (Nuroongji) found that texture had the greatest effect on preference [41,42]. However, in the study analyzing the preference for commercial ready-to-eat rice, the descriptive sensory analysis factor affecting the overall liking was the odor of grains, such as the odor of starch [30]. On the other hand, in general, in studies on food preference, taste and flavor are still reported to be the most important factors [43,44]. To summarize the results of previous studies and this study, the characteristics of texture and smell seem to be very important factors in the development of jellies or snacks using grains.

BJ_265, which contained 100% buckwheat flour, was closely associated with Brown_A, Soymilk_O, Buckwheat tea_O, Savory_F, plain flavor and taste, buckwheat flavor and taste, squashed texture, smooth texture, and stickiness among the 18 attributes. The BJ_453 sample (15% mung bean starch) showed a close relationship with the umami attribute.

## 4. Conclusions

Sensory attributes of buckwheat jelly (memilmuk) with mung bean flour added were determined using descriptive analysis. The 18 attributes were assessed; these included three appearance descriptors (brown, brightness, and roughness), four odor/aroma descriptors (soymilk smell, grain smell, red bean porridge smell, and buckwheat tea smell), six flavor or taste descriptors (savory flavor, plain taste, buckwheat taste, sweet taste, salty taste, and umami), and five texture descriptors (squashed, dry, smooth, elasticity, and stickiness). The results of the consumer acceptability test from 93 consumers showed a significant difference in harmony with the sauce, overall liking, willingness to try again, and intention to recommend attributes (*p* < 0.001). Among the six samples, buckwheat jelly with 25% mung bean starch (BJ_916) showed the highest score among consumers. The sample with 25% mung bean starch was closely related to a grain smell, elasticity, a red bean porridge smell, a sweet taste, and all attributes of consumer acceptability according to the results of PLSR analysis. These findings can be used to improve the organoleptic quality of buckwheat jelly and provide basic data to expand the consumer market due to its functional and sensory quality.

## Figures and Tables

**Table 1:** Ingredients, sample code, and cooking method of the six buckwheat jellies.

Sample Code	Percent (%) ^(1)^	Ingredient (g)					Cooking Method
		Buckwheat Flour	Mung Bean Powder	Water	Salt	Cooking Oil	
BJ_265	100:0	170.0	-	817.0	3.0	10.0	① Mix the ingredients, filter twice through a sieve (Chunggye Sanggong Co., Seoul, Korea), and rest at room temperature for 15 min. ② Put the mixture in a pot, put it on induction (Shinil Industrial Co., Ltd., Seoul, Korea) and stir at 180 °C for 5 min until it thickens, then reduce the temperature to 120 °C and stir for 7 min (stirring speed: 80 times/min). ③ Add cooking oil to Step 2, stir continuously for 5 min, turn off the heat, place it flat in a square mold, and solidify. ④ After 5 h at room temperature, cut into 3 cm × 1 cm × 1 cm pieces.
BJ_153	95:5	161.5	8.5				
BJ_870	90:10	153.0	17.0				
BJ_453	85:15	144.5	25.5				
BJ_335	80:20	136.0	34.0				
BJ_916	75:25	127.5	42.5				

^(1)^ Buckwheat flour and mung bean powder mixing ratio.

**Table 2:** Sensory attributes, definitions, and physical standards of buckwheat jelly.

Attributes (Descriptor)	Definition	Reference
Appearance		
Brown_A	Intensity of brown color	Formula guide (Pantone, Carlstadt, NJ, USA) ^(1)^
Brighness_A	Degree of brightness	Formula guide (Pantone, Carlstadt, NJ, USA)
Rougness_A	Degree of roughness	(Strong) Firm tobu (Pulmuone Co., Seoul, Korea) (13) ^(2)^ (Weak) Konjac jelly (Sajo Daelim Co., Seoul, Korea) (1)
Odor/Aroma		
Soymilk_O	The smell associated with soy milk	(Strong) sugar free soymilk (Maeil Dairies Co., Seoul, Korea) 25% + water 75% (15) (Weak) sugar free soymilk (Maeil Dairies Co., Seoul, Korea) 10% + water 90% (1)
Grain_O	The smell associated with toasted grain	(Strong) powder made of mixed grains (Damtuh Co., Sunheon, Jeollanam-do, Korea) 10% + water 90% (15) (Weak) powder made of mixed grains (Damtuh Co., Sunheon, Jeollanam-do, Korea) 1% + water 99% (1)
Red Bean Porridge_O	The smell associated with red bean porridge	(Strong) red bean porridge (Bibigo Porridge, CJ Cheiljedang Co., Seoul, Korea) 100% (15) (Normal) red bean porridge (Bibigo Porridge, CJ Cheiljedang Co., Seoul Korea) 50% + water 50% (8) (Weak) red bean porridge (Bibigo Porridge, CJ Cheiljedang Co., Seoul, Korea) 5% + water 95% (1)
Buckwheat tea_O	The smell associated with buckwheat tea in water	(Strong) buckwheat tea (Dongsuh Foods Co., Seoul, Korea) + 80 °C water 100 cc (15) (Weak) buckwheat tea (Dongsuh Foods Co., Seoul, Korea) + 80 °C water 1000 cc (3)
Flavor/Taste		
Savory_F	Intensity of flavor associated with boiled *Nurunggi*	(Strong) Nurungji Porridge (Ottogi Co., Ltd, Anyang, Gyeonggi-do, Korea) 100% (15) (Weak) Nurungji Porridge (Ottogi Co., Ltd, Anyang, Gyeonggi-do, Korea) 30% + water 70% (3)
Plain flavor and taste	Intensities of flavor and taste associated with plain soybean milk	(Strong) sugar free soymilk (Maeil Dairies Co., Seoul, Korea) 25% + water 75% (15) (Weak) sugar free soymilk (Maeil Dairies Co., Seoul, Korea) 5% + water 95% (1)
Buckwheat flavor and taste	Intensities of flavor and taste associated with buckwheat	(Strong) buckwheat tea (Dongsuh Foods Co.,Seoul, Korea) + 80 °C water 100 cc (15) (Weak) buckwheat tea (Dongsuh Foods Co.,Seoul, Korea) + 80 °C water 1000 cc (3)
Sweet taste	Typical taste of sucrose	(Strong) sugar 10% + water 90% (15) (Weak) sugar 1% + water 99% (1)
Salty taste	Typical taste of salt	(Strong) salt 0.5% + water 99.5 % (15) (Weak) salt 0.1% + water 99.9 % (1)
Umami	Typical taste of MSG (monosodium glutamate)	(Strong) MSG (Miwon, Daesang Co., Seoul, Korea) 1% + water 99 % (15) (Normal) MSG (Miwon, Daesang Co., Seoul, Korea) 0.5% + water 99.5 % (8) (Weak) MSG (Miwon, Daesang Co., Seoul, Korea) 0.1% + water 99.9 % (1)
Texture/Mouthfeel		
Squashed texture	Texture that crumbles when the sample is chewed (degree of squashed texture)	(Strong) sausage (Jinjuham Co., Seoul, Korea) (13)
Dry texture	Degree of dry texture associated with boiled egg yolk	(Strong) egg yolks boiled for 20 min (15) (Normal) egg yolks boiled for 10 min (10)
Smooth texture	Soft feel in the mouth (degree of smooth texture associated with soft tofu)	(Strong) soft tofu (Pulmuone Co., Seoul, Korea) (15) (Weak) firm tofu (Pulmuone Co., Seoul, Korea) (5)
Elasticity	Degree to which the sample returns to its shape when lightly pressed with the teeth in the mouth (degree of elasticity associated konjak jelly).	(Strong) konjac jelly (Sajo daelim Co., Seoul, Korea) (15) (Normal) sweet jelly of red beans (Haitai Confectionery and Foods Co., Seoul, Korea) (8)
Stickiness	Degree of stickiness and sticking to teeth when the sample is put in the mouth and eaten all (degree of stickiness associated with cheddar cheese)	(Strong) cheddar cheese (Maeil Dairies Co., Seoul, Korea) (15) (Weak) soft tofu (Pulmuone Co., Seoul, Korea) (3)

^(1)^ Indicates the brand used in this study. Other brands may be used to represent the attribute, but the reference intensities may need to be adjusted by the panel based on the intensity of that particular brand. ^(2)^ This number shows intensity of the standard reference.

**Table 3:** Intensities of sensory attributes of the six buckwheat jellies.

	Sample ^(1)^						*p*-Value ^(2)^
	BJ 265	BJ 153	BJ_870	BJ_453	BJ_335	BJ_916	
Appearance							
Brown_A	8.00 ^a (3)^	7.75 ^ab^	7.54 ^ab^	7.42 ^ab^	7.21 ^b^	7.25 ^b^	0.017
Brightness_A	8.38	8.38	8.38	8.46	8.33	8.29	0.999
Roughness_A	5.58	5.71	5.21	5.42	5.63	5.67	0.816
Odor/Aroma							
Soymilk_O	9.96 ^a^	9.19 ^ab^	8.90 ^b^	8.71 ^b^	8.81 ^b^	7.46 ^c^	<0.001
Grain_O	4.46	5.10	5.00	4.81	5.24	5.79	0.161
Red bean porridge_O	5.21 ^b^	5.90 ^ab^	5.48 ^b^	5.00 ^b^	5.76 ^b^	6.83 ^a^	0.006
Buckwheat tea_O	12.58 ^a^	12.33 ^a^	11.95 ^ab^	11.38 ^ab^	11.48 ^ab^	10.96 ^b^	0.046
Flavor/Taste							
Savory_F	4.42	4.08	4.08	3.83	3.79	3.63	0.626
Plain flavor and taste	6.67	6.29	5.92	6.00	6.00	5.5	0.414
Buckwheat flavor and taste	11.88	11.37	11.25	10.79	10.75	10.46	0.177
Sweet taste	2.58	2.54	2.63	2.21	2.38	2.92	0.394
Salty taste	3.63	3.08	3.08	3.00	3.04	3.17	0.365
Umami	2.29	2.58	2.46	2.67	2.33	2.42	0.995
Texture/Mouthfeel							
Squashed texture	12.92	12.42	11.75	11.46	11.29	11.00	0.052
Dry texture	7.25 ^a^	6.67 ^ab^	6.25 ^bc^	5.92 ^bc^	5.79 ^c^	6.42 ^bc^	<0.001
Smooth texture	10.71 ^a^	10.54 ^a^	9.79 ^ab^	9.38 ^ab^	9.35 ^ab^	8.63 ^b^	0.037
Elasticity	1.96 ^d^	2.17 ^cd^	2.63 ^bc^	2.83 ^ab^	2.80 ^ab^	3.38 ^a^	<0.001
Stickiness	10.79 ^a^	10.04 ^ab^	9.42 ^ab^	9.08 ^bcd^	8.83 ^cd^	8.17 ^d^	<0.001

^(1)^ Sample information: BJ 265 (0% mung bean powder), BJ 153 (5% mung bean powder), BJ_870 (10% mung bean powder), BJ_453 (15% mung bean powder), BJ_335 (20% mung bean powder) and BJ_916 (25% mung bean powder). ^(2)^ *p*-value by ANOVA. ^(3)^ Data were scored on a 15 point numerical scale, where 0 = no intensity of the attribute and 15 = extreme intensity of the attribute. ^a–d^ Different superscript letters mean significantly different among groups at α = 0.05 level by Duncan’s multiple range test.

**Table 4:** Consumer acceptability of the six buckwheat jellies ^(1)^.

	Sample ^(2)^												*p*-Value ^(3)^
	BJ_265		BJ_153		BJ_870		BJ_453		BJ_335		BJ_916		
	Mean	SD	Mean	SD	Mean	SD	Mean	SD	Mean	SD	Mean	SD	
Color liking	4.60	1.49	4.81	1.46	4.62	1.41	4.89	1.49	4.95	1.43	5.04	1.57	0.381
Smell liking	4.60	1.83	4.51	1.62	4.53	1.51	4.55	1.49	4.44	1.79	4.89	1.74	0.658
Savory taste liking	4.99	1.84	4.67	1.62	4.60	1.50	4.63	1.46	4.89	1.80	5.19	1.71	0.203
Aftertaste liking	4.59	1.96 ^b^	4.70	1.60 ^b^	4.32	1.54 ^b^	4.67	1.66 ^b^	4.86	1.75 ^b^	5.44	1.67 ^a^	0.004
Harmony with the sauce	4.44	1.83 ^c^	4.89	1.72 ^bc^	4.63	1.73 ^c^	5.26	1.67 ^ab^	5.34	1.73 ^ab^	5.70	1.66 ^a^	<0.001
Overall liking	4.15	1.82 ^c^	4.42	1.63 ^cd^	4.29	1.49 ^cd^	4.82	1.53 ^bc^	5.19	1.82 ^ab^	5.72	1.65 ^a^	<0.001
Recommend	3.30	1.78 ^d^	3.62	1.82 ^cd^	3.58	1.79 ^cd^	4.06	1.85 ^bc^	4.37	2.11 ^ab^	4.85	1.85 ^a^	<0.001
Try again	2.96	1.75 ^c^	3.10	1.78 ^c^	3.13	1.72 ^c^	3.80	1.85 ^b^	4.06	2.09 ^b^	4.68	1.89 ^a^	<0.001

^(1)^ Consumer acceptability test was conducted by 93 panelists using the nine-point hedonic scaling method (one point: dislike extremely, five points: moderate, nine points: like extremely). ^(2)^ Sample information: BJ 265 (0% mung bean powder), BJ 153 (5% mung bean powder), BJ_870 (10% mung bean powder), BJ_453 (15% mung bean powder), BJ_335 (20% mung bean powder) and BJ_916 (25% mung bean powder). ^(3)^ *p*-value by ANOVA. ^a–d^ Different superscript letters mean significantly different among groups at α = 0.05 level by Duncan’s multiple range test.

## Data Availability

Not applicable.
